# miR-382 inhibits tumor growth and enhance chemosensitivity in osteosarcoma

**DOI:** 10.18632/oncotarget.2418

**Published:** 2014-09-06

**Authors:** Meng Xu, Hua Jin, Cheng-Xiong Xu, Bo Sun, Zhi Mao, Wen-Zhi Bi, Yan Wang

**Affiliations:** ^1^ Department of Orthopaedics, The General Hospital of Chinese People's Liberation Army, Beijing, China.; ^2^ Department of Pharmaceutical Sciences, College of Pharmacy, University of South Florida, Tampa, FL, USA.; ^3^ Departments of Molecular Oncology, H. Lee Moffitt Cancer Center and Research Institute, Tampa, FL, USA.; ^4^ State Key Laboratory of Bioelectronics, School of Biological Science and Medical Engineering, Southeast University, Nanjing, China.

**Keywords:** miR-382, osteosarcoma growth, chemoresistance

## Abstract

Dysregulation of miRNAs is involved in osteosarcoma (OS). Here, we demonstrate that miR-382 is decreased in specimens of OS patients with a poor chemoresponse compared to those with a good chemoresponse. In addition, our clinical data show that decreased miR-382 was associated with poor survival in OS patients. Overexpression of miR-382 inhibited cell growth and chemoresistance by targeting KLF12 and HIPK3, respectively. In contrast, inhibition of miR-382 or overexpression of target genes stimulated OS cell growth and chemoresistance both *in vitro* and *in vivo*. Taken together, these findings suggest that miR-382 is a tumor suppressor miRNA and induction of miR-382 is a potential strategy to inhibit OS progression.

## INTRODUCTION

Osteosarcoma (OS) is the most common primary malignant tumor in children and adolescents [1]. The standard of care for appendicular OS in children leads to an overall 5-year survival rate of approximately 70% [2]. However, 30% of children diagnosed with OS will not survive for more than five years, and fewer than 50% will live beyond 10 years [3, 4]. A comprehensive understanding of OS biology is required to optimize treatment strategies and develop new chemotherapeutic agents [2]. Because treatment of this disease often fails due to chemoresistance development [5], it is especially important to analyze the molecular mechanisms that underlie this resistance. Such analyses could potentially lead to the development of novel treatment strategies for OS. The molecular basis of OS has received considerable attention during the past decade, and potential therapeutic targets are being identified. However, the cellular mechanisms that lead to the development of osteosarcoma and chemoresistance remain unclear.

MicroRNAs (miRNAs) are small non-coding RNA molecules of 18–22 nucleotides in length that regulate gene expression at the posttranscriptional level through interaction with complementary sequences in the 3′-UTRs of target mRNAs [6]. Differential miRNA expression between tumors and normal tissue has been described for many tumor types [2, 7], suggesting that miRNAs play an important role in tumorigenesis and disease progression. Indeed, recent studies show that aberrantly regulated miRNAs cause tumor initiation, development, metastasis and therapeutic resistance in many cancers by regulating multiple target genes [8, 9]. Studies also show that miRNA expression is dysregulated in OS tissue compared to normal bone. However, in contrast to other cancer types, little is known about the role of miRNAs in the pathogenesis of OS and regulation of abnormal gene expression [2, 10]. Recently, two studies reported that miR-382 levels were decreased in OS tissue or cell lines compared to normal bone [2, 11], suggesting that miR-382 may function as an onco-miRNA in OS. However, the effects on and molecular mechanism of miR-382 in OS are still unclear. Thus, we investigated the role and mechanism of miR-382 in OS.

## RESULTS

### Expression level of miR-382 in OS tissue closely correlated with clinical outcomes

**Figure 1:**
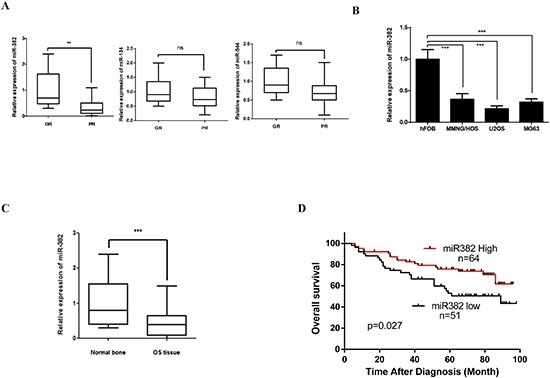
miR-382 expression in osteosarcoma (OS) tissue and OS cell lines **(A)** miR-382 expression significantly decreased in specimens of OS patients that had a poor chemoresponse (PR, n=44) compared to those with a good chemoresponse (GR, n=71). The data are presented as the mean ± SD. **(B)** OS cell lines show a low expression level of miR-382 compared to human fetal osteoblastic (hFOB) cells. The data are presented as the mean ± SD from three independent experiments. **(C)** miR-382 expression was decreased in OS tissue (n=115) compared to normal bone (n=107). The data are presented as the mean ± SD. **(D)** Kaplan-Meier survival rates for OS patients with low (n=51) and high (n=64) miR-382 expression *, *p*<0.05; **, *p*<0.01; ***, *p*<0.001.

Previous study show that several members of the 14q32 locus miRNAs including miR-382, miR-134, and miR-544 were inversely correlated with OS patients clinical outcomes [12]. However, our data show only miR-382 expression was significantly decreased in specimens from OS patients that showed a poor chemoresponse compared to those that responded well to chemotherapy (Figure 1A). So, in this study, we focused on miR-382 function in OS progression. Similar with previous report, our data also show that the expression of miR-382 was significantly decreased in OS cell lines and OS tissues compared to human fetal osteoblastic cells (hFOB) and normal bones (Figures 1B and C). Furthermore, our clinical results show that expression of miR-382 was inversely correlated with OS patient survival (Figure 1D). These results suggest that miR-382 may be plays an important role in OS development and chemoresistance.

### Decreased expression of miR-382 contributes to OS growth in vitro and in vivo

**Figure 2:**
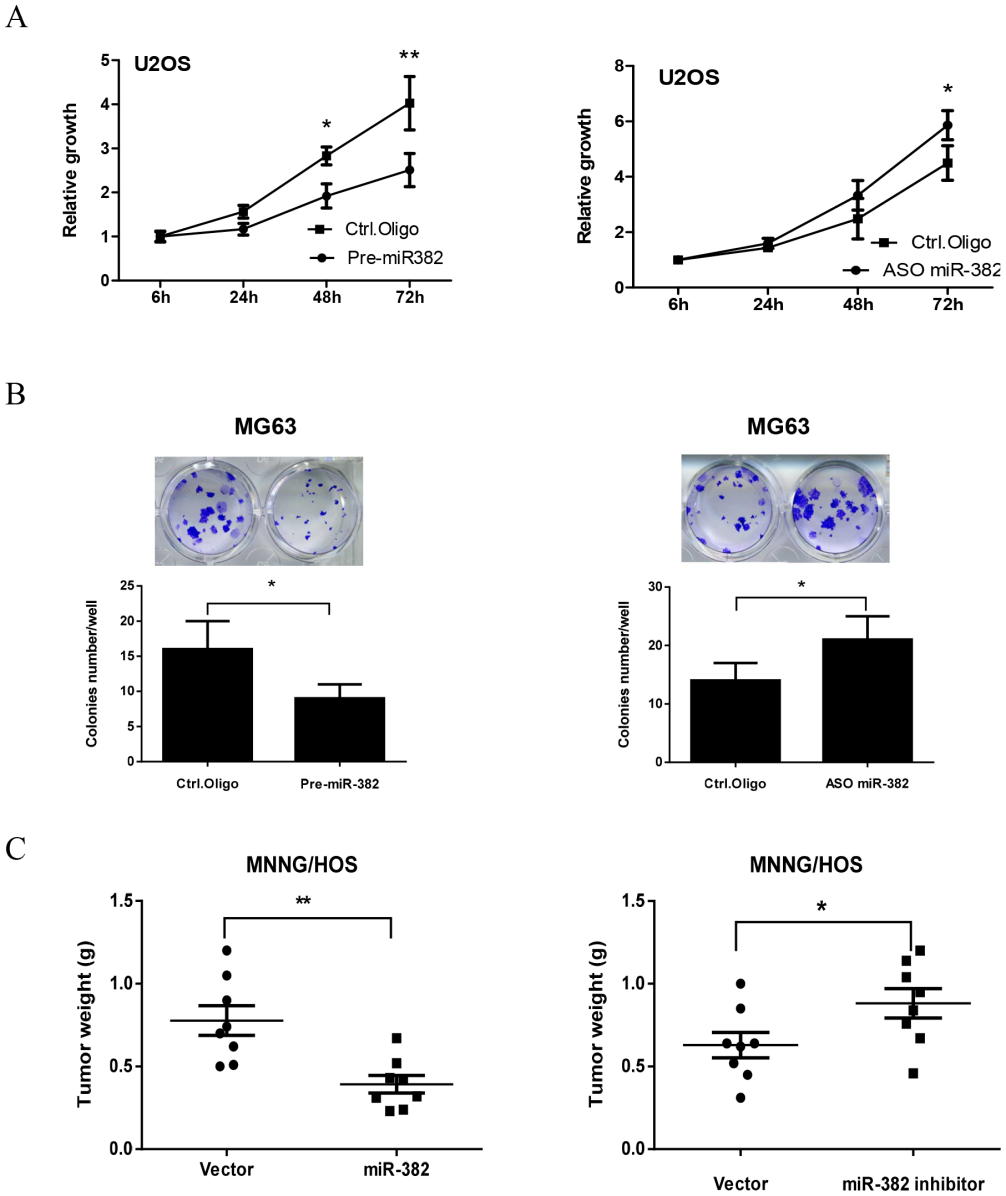
miR-382 suppresses osteosarcoma (OS) growth *in vitro* and *in vivo* **(A)** Overexpression of miR-382 inhibits U2OS proliferation, whereas inhibition of miR-382 stimulated U2OS proliferation compared to the control. U2OS cells were transfected with control oligonucleotides (Ctrl. Oligo), pre-miR-382 or antisense oligonucleotides of miR-382 (ASO miR-382). After 24 hrs of transfection, cells were seeded into 96-well culture plates at a density of 4 × 10^3^ cells/well. Cell proliferation was subsequently assessed by an MTT assay at the indicated time. **(B)** Overexpression of miR-382 inhibited colony formation, whereas inhibition of miR-382 stimulated colony formation compared to the control in MG63 cells. MG63 cells were transfected with indicated nucleotides. After 24 hrs of transfection, 200 cells/well were seeded in 24-well plates and the medium was changed every 3 days. After 12 days, the plates were stained for the formation of cell colonies with crystal violet dye and the number of colonies was counted. **(C)** Overexpression of miR-382 inhibited tumor growth, whereas inhibition of miR-382 stimulated tumor growth in a MNNG/HOS xenograft model. MNNG/HOS cells were transfected with the indicated plasmid. After 36 hrs of transfection, 2 × 10^6^ cells in serum-free medium were injected s.c. into nude mice (n = 8/group). One month after cell injection, mice were sacrificed and the tumors were weighed. *, *p*<0.05; **, *p*<0.01 compared to control.

Next, we investigated the effects of miR-382 on OS cell growth by overexpression or inhibition of miR-382 (Supplementary Figure S1). As shown in Figure 2A, overexpression of miR-382 significantly inhibited U2OS cell growth. In contrast, inhibition of miR-382 stimulated U2OS cell growth (Figure 2A). We further confirmed these results with a clonogenic assay using another OS cell line, MG63. Overexpression of miR-382 inhibited MG63 colony formation, whereas inhibition of miR-382 stimulated MG63 colony formation compared to the control (Figure 2B). These results parallel those obtained from MTT assays performed on U2OS cells. Finally, we confirmed these *in vitro* results using a MNNG/HOS xenograft model. As shown Figure 2C, overexpression or knockdown of miR-382 inhibited or stimulated tumor growth, respectively, compared to the control group.

### Decreased expression of miR-382 contributes to chemoresistance in OS

**Figure 3:**
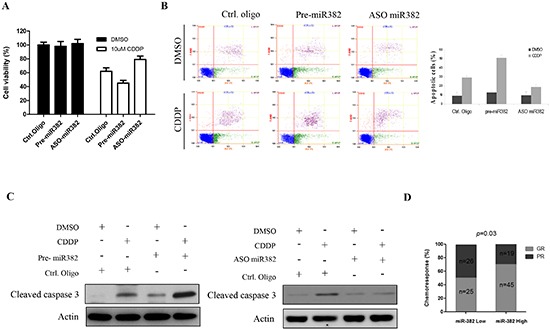
miR-382 enhanced CDDP-induced apoptosis in osteosarcoma (OS) cell lines **(A)** Overexpression of miR-382 stimulates CDDP-induced U2OS cell death, whereas inhibition of miR-382 protected U2OS cells form CDDP-induced death. U2OS cells were transfected with indicated nucleotides. After 24 hrs of transfection, 4 × 10^3^ cells/well were seeded in 96-well cell culture plates. The next day, cells were incubated with or without the indicated concentration of CDDP for 48 hrs and subsequently subjected to an MTT assay. The data are presented as the mean ± SD from three independent experiments. **(B)** Overexpression of miR-382 increased CDDP-induced apoptosis in MG63 cells. In contrast, inhibition of miR-382 inhibited CDDP-induced apoptosis in MG63 cells. MG63 cells were transfected with the indicated nucleotides. After 24 hrs of transfection, cells were seeded into 6-well cell culture plates. The next day, cells were treated with DMSO or 10 μM CDDP for 24 hrs, followed by a flow cytometric assay. The data are presented as the mean ± SD from three independent experiments. **(C)** Overexpression of miR-382 increased CDDP-induced caspase 3 cleavage, whereas inhibition of miR-382 suppressed CDDP-induced caspase 3 cleavage in U2OS cells. **(D)** High miR-382 patients more sensitive to chemotherapy compared to low miR-382 patients.

As shown above, miR-382 was significantly decreased in specimens of OS patients that had a poor chemoresponse compared to those that responded well to chemotherapy, suggesting that miR-382 may be involved in OS chemoresistance. Thus, we examined the effects of miR-382 on chemotherapeutic sensitivity in OS using CDDP, doxorubicin, and MTX because these three drugs are commonly used anticancer drugs in OS[13]. As expected, inhibition of miR-382 protected cells from CDDP-induced death in U2OS cells, whereas overexpression of miR-382 increased cell death induced by CDDP (Figure 3A). These results were further confirmed in MG63 cells by flow cytometric analysis. The flow data show that overexpression of miR-382 enhanced CDDP-induced cell apoptosis, whereas inhibition of miR-382 significantly reduced the number of apoptotic cells induced by CDDP (Figure 3B). Consistent with these data, Western blotting data also show that expression or knockdown of miR-382 increased or decreased CDDP-induced cleavage of caspase 3, respectively (Figure 3C). In addition, we examined the effect of miR-382 on OS cell chemosensitivity to doxorubicin and MTX. As with the CDDP experiment, overexpression of miR-382 increased doxorubicin- or MTX-induced cell death by increasing apoptosis, whereas silencing of miR-382 protected cells from these drugs (Supplementary Figure S2). Finally, our clinical data show that patients with high miR-382 more sensitive to chemotherapy compared to patients with low miR-382 (Figure 3D).

### KLF12 and HIPK3 are targets of miR-382

**Figure 4:**
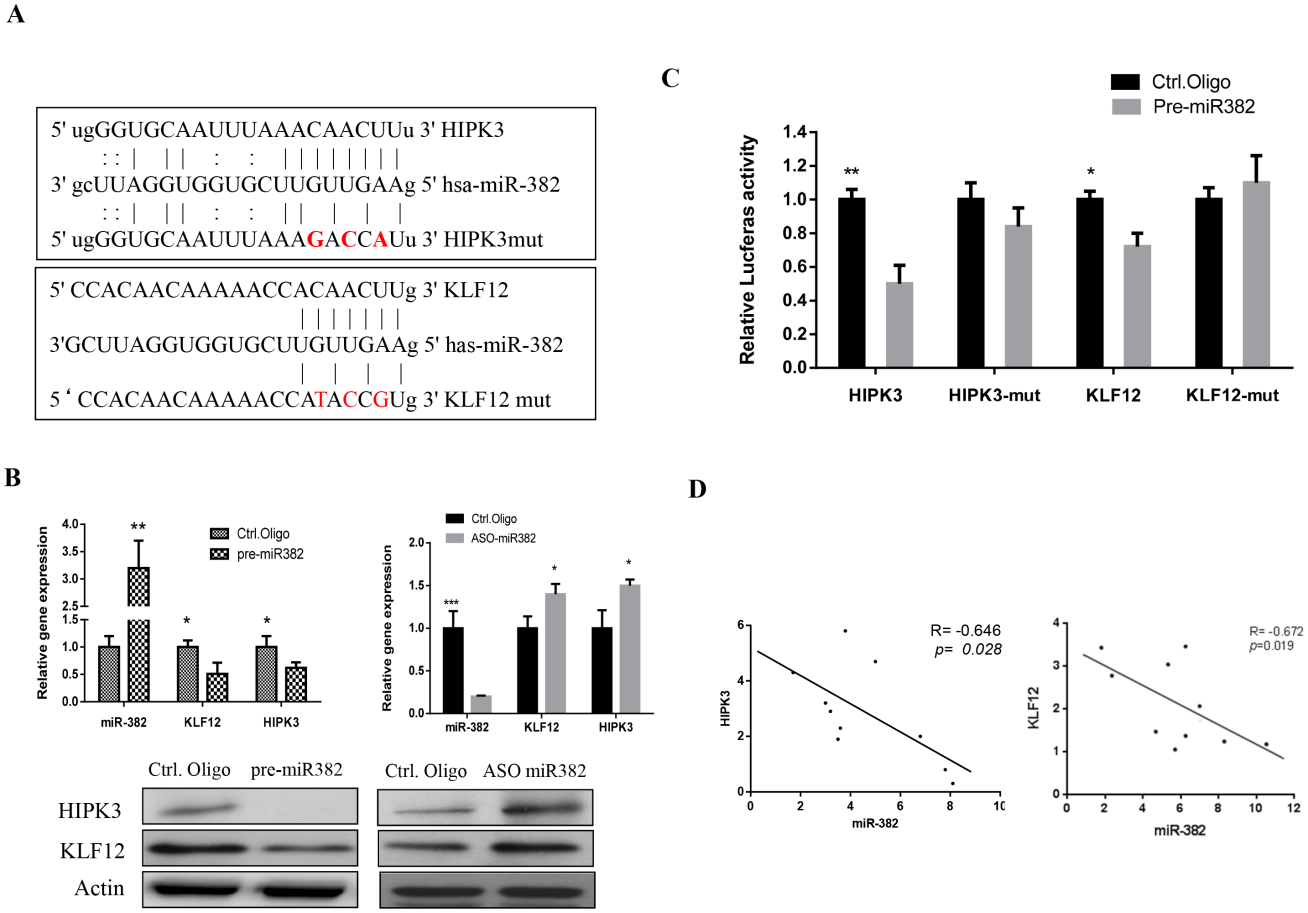
KLF12 and HIPK3 are targets of miR-382 **(A)** Sequence alignment of miR-382 with the 3′ UTR of the KLF12 and HIPK3 genes. **(B)** Expression of KLF12 and HIPK3 was negatively regulated by miR-382 at both mRNA and protein levels. U2OS cells were transfected with the indicated nucleotides. After 3 d of transfection, cells were harvested and their mRNA was isolated for q-RT PCR analysis while their proteins were harvested for Western blot analysis. **(C)** 3′UTR luciferase reporter assay for KLF12 and HIPK3. HEK293T cells were cotransfected with a indicated pMIR-3′UTR luciferase reporter construct and pre-miR-382 or respective controls (Ctrl. Oligo). After 36 hrs of transfection, luciferase intensity was assessed. The data are presented as the mean ± SD from three independent experiments. **(D)** The inverse correlation between miR-382 and KLF12 or HIPK3 mRNA expression in osteosarcoma samples (n=10) by linear regression analysis. *, *P* ≤ 0.05

miRNAs function as negative regulators of gene expression regulation. Here, we found that KLF12 and HIPK3 were tentative targets of miR-382 (Figure 4A). As shown in Figure 4B, KLF12 and HIPK3 expression were significantly decreased by ectopic over expression of miR-382 at both the mRNA and protein level. In contrast, inhibition of miR-382 increased the expression of KLF12 and HIPK3 at both the mRNA and protein level. To determine whether the KLF12 and HIPK3 luciferase expression regulation depended on the binding between their complementary 3′ UTR sequences and the miR-382 seed, a three point mutation was inserted in the KLF12 or HIPK3 3′ UTR, as indicated in Figure 4A. The results show that miR-382 overexpression significantly repressed luciferase activity in the wild-type 3′ UTR treatment. In contrast, both 3′ UTR alterations completely abrogated the effect of miR-382 overexpression on luciferase expression in HEK293T cells (Figure 4C). In addition, we observed an inverse correlation between the expression of miR-382 and KLF12 or HIPK3 in OS patients (Figure 4D). Taken together, these data suggest that miR-382 negatively regulates the expression of KLF12 and HIPK3 by directly targeting their 3′ UTR sequences.

### KLF12 and HIPK3 stimulate cell growth and chemoresistance in OS cells, respectively

**Figure 5:**
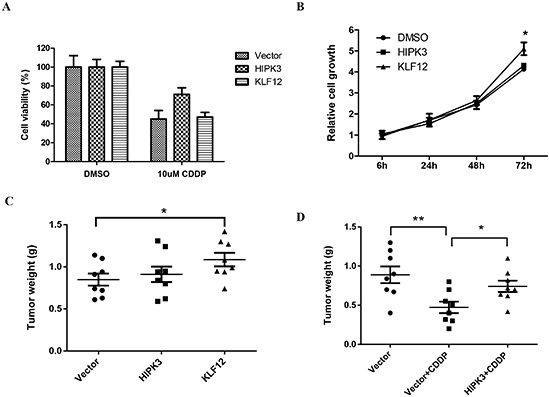
HIKP3 and KLF12 stimulate chemosensitivity and OS cell growth, respectively **(A)** Overexpression of HIKP3 protected cells from death induced by CDDP treatment. KLF12 overexpression did not have this effect. MG63 cells were transfected with the indicated plasmid. After 24 hrs, cells were seeded into 96-well cell culture plates. The next day, cells were treated with DMSO or 10 μM CDDP for 48 hrs and cell viability was measured with a MTT assay. The data are presented as the mean ± SD from three independent experiments. **(B)** Overexpression of KLF12, but not HIKP3, stimulated cell growth. MG63 cells were transfected with the indicated plasmid. After 24 hrs, cells were seeded into 96-well cell culture plates and cell viability was measured at the indicated time using a MTT assay. The data are presented as the mean ± SD from three independent experiments. **(C)** In the MNNG/HOS xenograft model, tumor growth was increased by KLF12 overexpression, but not by HIPK3 overexpression. **(D)** HIPK3 overexpression induced resistance to CDDP in the MNNG/HOS xenograft model.

To determine whether the anti-tumor effects of miR-382 on these OS cells could be partly explained by its targeting of KLF12 and HIPK3, we first analyzed how KLF12 and HIPK3 overexpression affected *in vitro* and *in vivo* cell growth and chemosensitivity. Our data show that the overexpression of HIPK3 protected MG63 cells from CDDP-induced death. Overexpression of KLF12 did not have effect (Figure 5A). However, KLF 12 overexpression significantly stimulated MG63 cell growth compared to the vector control and HIPK3 overexpression group (Figure 5B). Furthermore, we confirmed these results in an MNNG/HOS xenograft model. The *in vivo* results parallel the *in vitro* results and show that KLF12 and HIPK3 overexpression stimulate tumor growth and chemoresistance, respectively (Figures 5C and D).

### miR-382-mediated expression of KLF12 and HIPK3 controls miR-382 function

**Figure 6:**
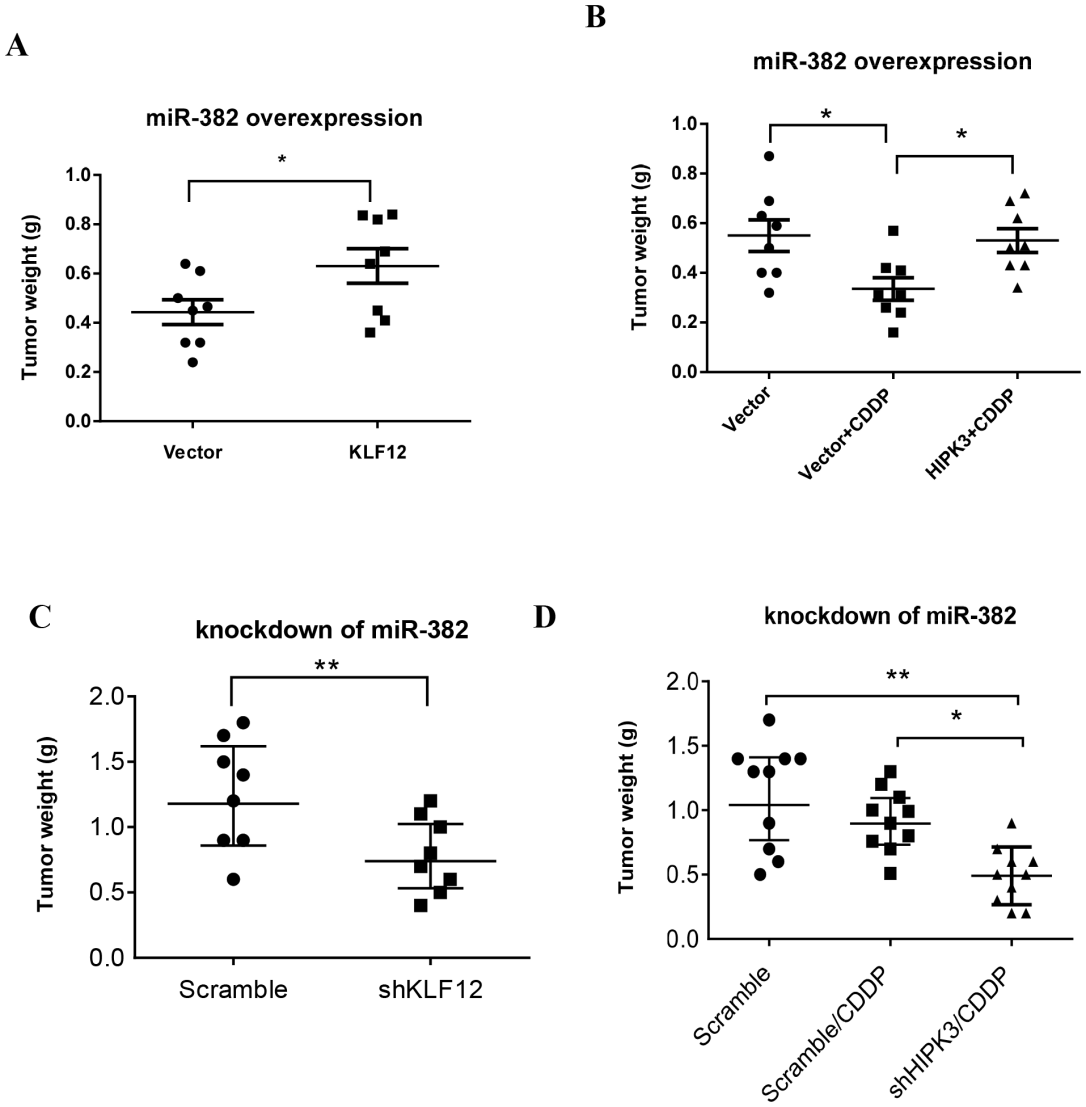
KLF12 and HIPK3 are involved in the function of miR-382 **(A)** KLF12 overexpression stimulated tumor growth in miR-382-overexpressing a MNNG/HOS xenograft model. **(B)** Overexpression of HIPK3 induced resistance to CDDP in a miR-382-overexpressing MNNG/HOS xenograft model. **(C)** Knockdown of KLF12 inhibited tumor growth in a miR-382-silenced MNNG/HOS xenograft models. **(D)** Knockdown of HIPK3 enhanced chemosensitivity to CDDP in a miR-382-silenced MNNG/HOS cell xenograft model.

As presented above, miR-382 can modulate the cell growth-related gene KLF12 and the chemosensitivity-related gene HIPK3. Hence, we sought to determine whether the anti-tumor phenotype associated with miR-382 overexpression could be rescued by KLF12 or HIPK3 overexpression in miR-382-overexpressing cells. For this purpose, an expression of KLF12 or HIPK3 construct lacking its respective 3′ UTR was transiently transfected into cells that stably overexpress miR-382 (MNNG/HOS) (Supplementary Figures S3A and B). As shown in Figures 6A and B, overexpression of KLF12 and HIPK3 significantly stimulated tumor growth and chemoresistance in miR-382-overexpressing MNNG/HOS cell xenograft models, respectively. In contrast, knockdown of KLF12 and HIPK3 respectively inhibited tumor growth and enhanced chemosensitivity in a miR-382-knockdown MNNG/HOS cell xenograft model (Figures 6C and D). In conclusion, these experiments first provided that KLF12 and HIPK3 are major functional downstream players of miR-382 in OS.

### The prognostic significance of miR-382 for survival is due to the potential effect on chemoresistance

We next analyzed factors predictive of poor overall survival in OS patients using univariate and multivariate Cox regression analysis (Supplementary Table 2). In the univariate analysis, poor survival in OS patients was associated with miR-382 expression level and chemoresistance. In multivariate analysis, the chemoresponse is still significantly associated with survival (*p*=0.02), however, we did not observed significant correlation between the miR-382 expression level and survival (*p*=0.07). In addition, above data show that miR-382 significantly affects chemoresponse in OS, suggesting that miR-382 effects on OS patients may be due to affect chemoresistance. So, we next performed Spearman correlation analysis. As expected, our clinical data show that the expression of miR-382 is significantly correlated to chemoresponse in OS patients (*p*=0.01, correlation coefficient = - 0.24).

## DISCUSSION

Our knowledge of the molecular pathogenesis of OS has increased tremendously during the past decade. However, a comprehensive understanding of the molecular mechanisms of the disease, such as its tumorigenesis, specific mediators of disease progression, occurrence of chemoresistance, and development of metastasis [14], remains elusive. Recent studies have shown that dysregulated miRNAs significantly contribute to all stages of OS progression by regulating their target genes [15, 16]. Interestingly, individual miRNAs may have dual functions in tumor progression as either a tumor promoter or suppressor [17-19]. Accordingly, several studies suggest that miR-382 may also play dual role in different cancer types. Significantly increased expression of miR-382 was identified in multiple myeloma [20] and acute myeloid leukemia with common translocations [21], and the authors indicated that increased miR-382 may contribute to the development of these diseases. However, in current study, we provide evidence that miR-382 acts as a cancer suppressor in OS. Importantly, inhibition of miR-382 or overexpression of its target genes in OS cells enables them develop chemoresistance and proliferate. Conversely, miR-382 overexpression or concomitant miR-382 silencing and target gene knockdown impairs the establishment of an aggressive phenotype, suggesting a major role for miR-382 and its downstream target genes in controlling OS progression. In agreement with our findings, Thayanithy et al. reported decreased miR-382 levels in OS cell lines compared to normal bone and an anticancer effect on OS cells by miR-382 through the targeting of cMyc [22]. Taken together, these data suggest that miR-382 suppresses OS tumors.

Although significant advances have been made in the treatment of OS, chemoresistance constitutes a persistent problem in the treatment of this disease. CDDP, doxorubicin, and MTX are commonly used anticancer drugs in OS [13]. Patients who do not respond to these drugs have a poor prognosis [5]. However, the mechanism of chemoresistance in OS is still unclear. Our data present the first evidence that miR-382 is markedly downregulated in tumors of OS patients that have a poor chemoresponse compared to those that respond well to chemotherapy. Furthermore, we have found that experimental restoration of miR-382 expression in OS cells leads to enhanced chemosensitivity to anti-cancer drugs, whereas complete inhibition of miR-382 promoted chemoresistance. In addition, our *in vivo* data show that concomitant miR-382 inhibition and target gene knockdown increases chemosensitivity. This effect was not observed when the target genes lacked their 3′ UTR sequences. Taken together, these findings suggest that miR-382 and its downstream target genes play important roles in controlling OS chemosensitivity.

As described above, miRNAs play different roles by targeting different genes. Thus, investigating the target genes remains important to understand the molecular mechanisms by which a miRNA promotes or suppresses oncogenesis. In this study, we identified KLF12 and HIKP3 as miR-382 target genes. Our data show that restoration of miR-382 expression in OS cells leads to suppression of KLF12 and HIKP3, whereas inhibition of miR-382 further upregulates KLF12 and HIKP3. Our luciferase reporter experiments show that miR-382 directly targets the 3′ UTR of KLF12 and HIPK3. Furthermore, an inverse correlation between the miR-382 and KLF12 or HIKP3 expression levels was demonstrated in human OS samples. Nakamura et al. reported that KLF12 acts as an oncogene and plays an important role in gastric cancer progression by stimulating cancer cell growth [23]. In fact, increased expression of KLF12 was also detected by microarray gene profiling in OS tissue, but its role was unclear [24, 25]. Our data present the first evidence for KLF12's strong stimulation of OS cell growth *in vitro* and *in vivo*. We also demonstrated that the expression of KLF12 was controlled by miR-382. Importantly, overexpression of KLF12 increased tumor growth in a miR-382-overexpressing MNNG/HOS xenograft model. Conversely, knockdown of KLF-12 inhibited tumor growth in a miR-382-knockdown MNNG/HOS xenograft model. These data suggest that downregulation of miR-382 can stimulate cancer growth by targeting KLF12 in OS. In this study, we also discovered that HIPK3 is a target of miR-382. Previous studies have shown that HIPK3 is implicated in the multidrug resistance of a number of tumors [26-28]. Consistent with these reports, our data also show that overexpression of HIPK3 induces CDDP resistance *in vitro* and *in vivo*. More importantly, overexpression of HIPK3 induced chemoresistance in miR-382-overexpressing OS cells, whereas knockdown of HIPK3 improved chemosensitivity to CDDP in miR-382-knockdown OS cells. These data suggest that decreased miR-382 contributes to OS growth and chemoresistance by targeting the KLF12 and HIPK3 genes, respectively.

In this study, we identified a significantly decreased expression level of miR-382 in OS cells and patient tumor samples. In addition, our results show that the decreased expression of miR-382 closely correlated with poor chemosensitivity and poor survival in OS patients. In agreement with our findings, Sarver et al. also demonstrated an inverse correlation between the miR-382 expression level and positive clinical outcomes using small-scale human OS samples, suggesting that miR-382 may be a useful prognostic and predictive marker candidate that can assist in the management of patients with OS. However, our multivariable analysis results show that miR-382 unlike chemoresistance no correlation with poor survival in OS patients. These data suggesting that the prognostic significance of miR-382 for survival may be due to the potential effect on chemoresistance. In fact, clinical data show that the expression level of miR-382 is significantly correlated to chemoresponse in OS patients.

The survival of patient with OS has markedly improved from less than 20% reported in the 1960s [29-31] to approximately 65-70% in the 1970s [32]. However, the survival of OS patients has remained unchanged over the past 30 years. Treatment of OS often fails due to chemoresistance development. Recent studies show that chemoresistance in OS appears to be mediated by numerous mechanisms including perturbations in signal transduction pathways and microRNA (miRNA) dysregulation [33]. In addition, studies show targeting these essential pathways in combination with conventional therapy could be a strategy to improve the efficacy of current drug regimens in OS [34-36]. In this study, our data show overexpression of miR-382 or knock down of target genes can significantly inhibits OS cell growth and chemoresistance both *in vitro* and *in vivo*. Taken together, these findings suggest that induction of miR-382 is a potential strategy to overcome drug resistance in OS.

In summary, we combined clinical and experimental studies to establish a role for miR-382 in OS. Our work led to the identification of a novel functional pathway controlled by miR-382 and its direct targets, KLF12 and HIPK3, that coordinate tumor growth and chemosensitivity, respectively, in OS. Therefore, miR-382 and its target gene pathway may represent new therapeutic opportunities for chemoresistant OS.

## MATERIALS AND METHODS

### Reagents

3-(4,5-dimethylthiazol-2-yl)-2 and 5-diphenyltetrazolium bromide (MTT), puromycin, cisplatin (CDDP), methotrexate (MTX), anti-caspase 3 antibody, anti-actin antibody and cell culture meium were purchased from Sigma. A miR-382 expression vector, inhibitor (vector system), shRNA of KLF12, KLF12 expression plasmid, shRNA of HIPK3 and HIPK3 expression plasmid were obtained from GeneCopoeia™. An anti-HIPK3 antibody and anti-KLF12 antibody were purchased from Santa Cruz and Abcam, respectively. An apoptosis assay kit was purchased from Biotium Inc. A dual luciferase assay kit and lipofectamine 2000 were obtained from Promega and Invitrogen, respectively. Trizol, OPTI MEM, pre-miR-382 and antisense nucleotides of miR-382 were purchased from Life technologies.

### Cell culture and human specimens

MNNG/HOS, U2OS and MG63 cells were purchased from the American Type Culture Collection (Manassas, VA) and cultured in RPMI1640, Eagle's Minimum Essential Medium and Dulbecco's modified Eagle's medium supplemented with 10% fetal bovine serum, respectively.

Human specimens were obtained from diagnostic biopsies for analysis of miR-382 expression. A total 115 of patients specimens were used in this study and no metastasis was found in these patients at diagnosis. Specimens were gently washed with normal saline to remove excess blood, and placed immediately into liquid nitrogen and 10% formalin solution by the surgeon. The patients' characteristics at the original diagnosis are summarized in Supplementary Table 1, and there was no significant difference between the two groups with respect to their general characteristics. This research was approved by the Research Ethics Board of the General Hospital of the People's Liberation Army.

### Histologic response evaluation

All patients were received same multidrug chemotherapy. After preoperative chemotherapy, the tumors resected and an expert panel of pathologists were reviewed the histologic response. When the percentage of tumor necrosis was ≥90%, the patients were classified as good responders, and when the percentage of tumor necrosis was lower than 90%, the patients were defined as poor responders [37].

### Cell proliferation assay

Cells were transfected with the indicated plasmid or nucleotides using lipofectamine 2000. After 24 hrs of transfection, the cells were seeded into 96-well culture plates at a density of 4 × 10^3^ cells/well. Cells were incubated with the indicated drug for an indicated time. The cell proliferation was measured using MTT according to the manufacturer's protocol.

### Clonogenic assay

For the clonogenic assay, cells were seeded into 24-well cell culture plates at a density of 200 cells/well. Cells were subsequently incubated with or without CDDP, and this treatment was repeated every 3 d. After 12 d, the plates were stained for the formation of cell colonies with crystal violet in 70% ethanol and counted under the microscope. The colony forming efficiency was calculated from the number of colonies (consisting of at least 50 cells).

### Apoptosis analysis

After 6 hrs of transfection, cells were seeded in 6-well cell culture plates. Cells were incubated with indicated drugs. 24 hrs later, cells were harvested, then, stained with annexin V and 7-aminoactinomycin D (7-AAD) according to the manufacturer's protocol, followed by flow cytometric analysis.

### Real-time quantitative PCR analysis

For the qRT-PCR analysis, the total RNA was isolated from OS cells or human specimens using the TRIzol reagent (Invitrogen) according to the manufacturer's protocol. For miR-382 and RNU6B, reverse transcription and real-time PCR were performed with The TaqMan MicroRNA reverse transcription kit and TaqMan Universal PCR Master Mix, respectively, using Ambion miRNA primers. miRNAs expression level was normalized to RNU6B.

For other gene expression, reverse transcription and PCR were performed with a High Capacity cDNA Reverse Transcription Kit (Life technologies) and QuantiTect SYBR Green PCR kit (Qiagen), respectively. The primer sequences for genes were defined as follows: KLF12 forward 5′-CACCTGGAAATG TGAACAACA-3′, reverse 5′-TTTTACTTTGTCTGGGAGATAGGC-3′; HIPK3 forward 5′-ACATTGGAAGAGCATGAGGCAGAGA-3′, reverse 5′-CTGCTG AAAAGCATCACCACAACCA-3′; GAPDH forward 5′-GCAGGGGGGAGCCAAAA GGGT-3′ and reverse 5′-TGGGTGGCAGTGATGGCATGG-3′.

### Luciferase reporter assay

The 3′-UTR segments of KLF12 and HIPK3 that were predicted to interact with miR-382 were amplified by PCR from human genomic DNA and inserted into the *Mlu*I and *Hin*dIII sites of the miRNA Expression Reporter Vector. For the luciferase reporter experiments, the indicated cells were seeded into 24-well cell culture plates and transfected with the indicated reporter plasmids containing firefly luciferase and either the pre-miR-382 or control oligonucleotides (Ctrl.Oligo) or an antisense nucleotides of miR-382 (ASO miR-382). These cells were also cotransfected with a Renilla luciferase plasmid as an internal control. Following 48 h of incubation, cells were subjected to a luciferase reporter assay. Luciferase activity was measured using the dual-luciferase assay system according to the manufacturer's protocol. The luciferase activity was normalized by the activity of renilla luciferase. Each experiment was repeated at least three times in triplicate.

### Western blot analysis

Thirty micrograms of protein were separated by sodium dodecyl sulfate-polyacrylamide gel electrophoresis and transferred onto nitrocellulose membranes. The membranes were blocked with 5% non-fat milk for 1 h and incubated overnight at 4 °C with their corresponding primary antibodies (1:1000 dilution in 3% BSA), followed by incubation with secondary antibodies conjugated to horseradish peroxidase (HRP) (1:10000 dilution in 5% non-fat milk) for 3 hrs at RT.

### Stable cell line selection

MNNG/HOS cells were transfected with miR-382 expression vector or antisense-miR-382 expression vector. After 36 hrs of transfection, cells were incubated with 2μg/ml puromycin. The expression of miR-382 was measured after 7 days of treatment with puromycin and frozen down in aliquots for later use.

### Animal experiments

To investigate the effects of the indicated genes in tumor growth, MNNG/HOS cells were transfected with a corresponding plasmid. After 36 hrs of transfection, 2 × 10^6^ cells in serum-free medium were injected s.c. into six-week old female athymic (nu/nu) mice (n = 8/group). The mice were sacrificed 1 month after cell injection, and the tumor weights were measured.

For the chemotherapy experiment, MNNG/HOS or stable MNNG/HOS (overexpressing miR-382 or knockdown miR-382) were transfected with the indicated plasmid. After 36 hrs of transfection, 2 × 10^6^ cells in serum-free medium were injected s.c. into six-week old female athymic (nu/nu) mice (n = 8/group). When tumors reached a size of approximately 100 mm^3^, the mice were started on a treatment of either PBS or CDDP (10 mg/Kg body weight). The treatment was administered every third day. After 3 weeks. The mice were sacrificed and the tumor weights were measured.

### Statistic analysis

The survival rate of OS patients was calculated using Kaplan-Meier survival analysis. Differences between groups were determined by an unpaired Student's *t*-test or one-way ANOVA method using the SAS statistical software package version 6.12 (SAS Institute, Cary, NC). A *p*-value less than to 0.05 was considered to be statistically significant.

## SUPPLEMENTARY FIGURES AND TABLES


