# Supplementary material for: miR-382 inhibits tumor growth and enhance chemosensitivity in osteosarcoma
Source: Oncotarget. 2014 Sep 6;5(19):9472–83. doi: 10.18632/oncotarget.2418 (PMC4253447; doi:10.18632/oncotarget.2418)
Supplement: Supplementary file 1 [file oncotarget-05-9472-s001.pdf]

SUPPLEMENTARY FIGURES AND TABLES

A

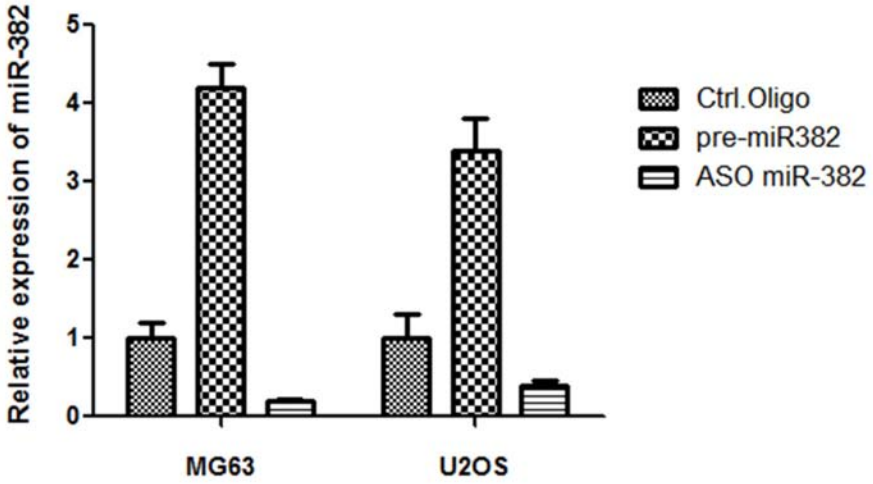

B

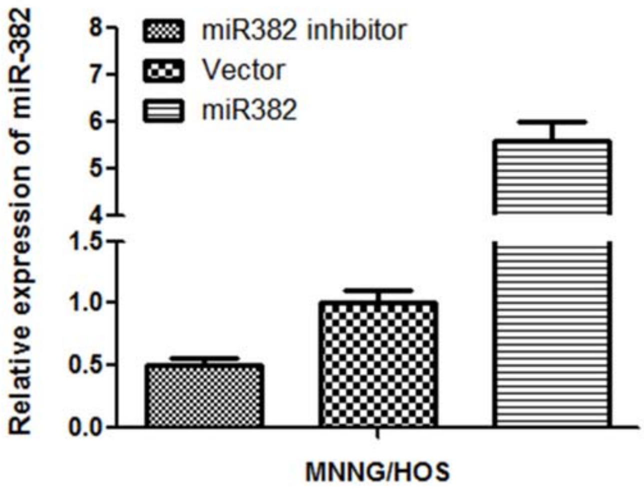

Supplementary Figure S1. The expression of miR-382 was measured by q-RT PCR. (A) Indicated cells were transfected with control oligonucleotide (Ctrl. Oligo) or pre-miR-382 or antisense oligonucleotide of miR-382 (ASO miR-382). After 72 hrs of transfection, isolated mRNAs, then subjected to q-RT PCR. **(B)** MNNG/HOS cells were transfected with miR-382 expression vector or miR-382 inhibitor (vector system). After 72 hrs of transfection, isolated mRNAs, then subjected to q-RT PCR. All data are presented as the mean ± SD from three independent experiments.

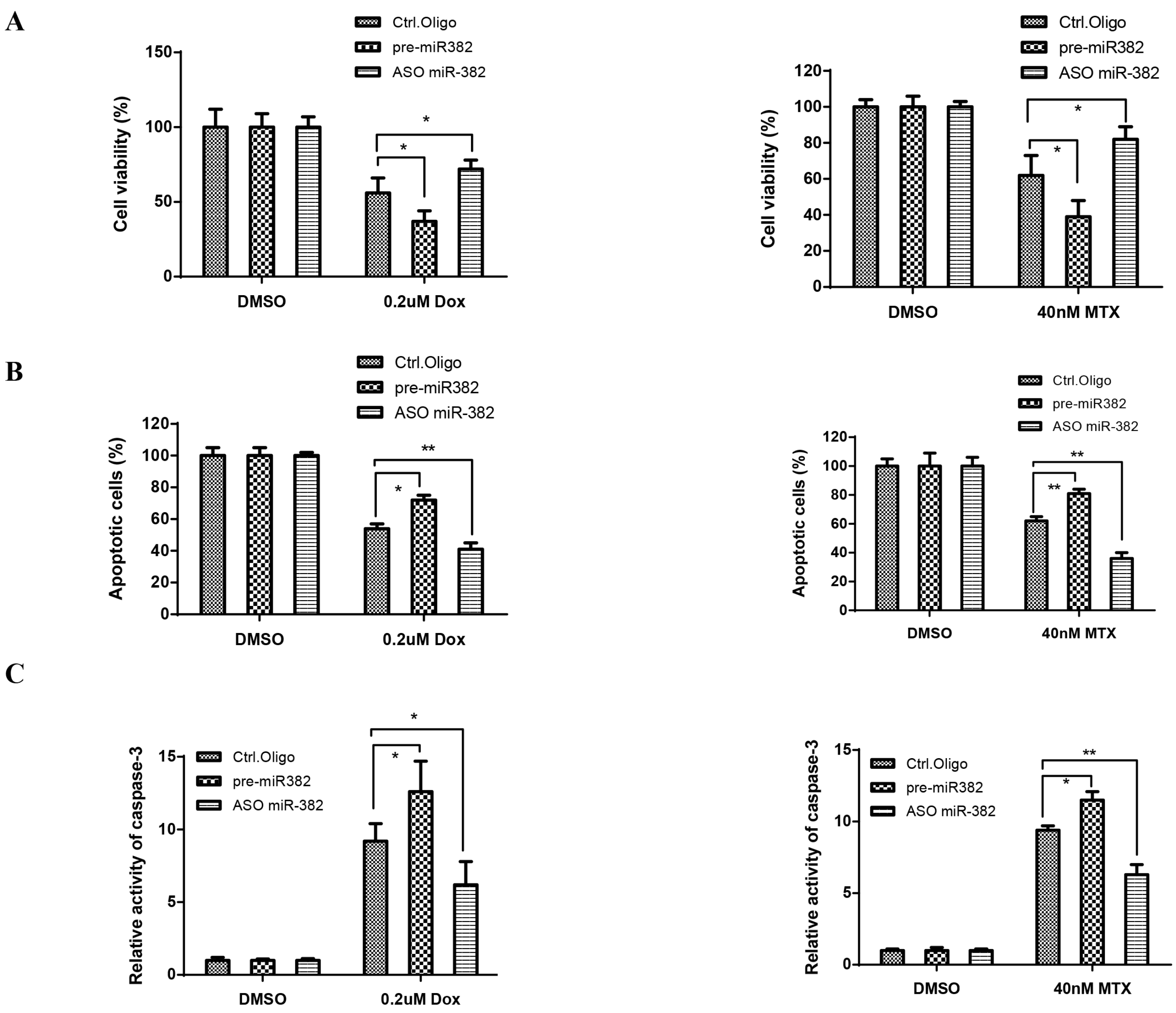

Supplementary Figure S2. miR-382 enhanced doxorubicin- or MTX-induced apoptosis in OS cell lines. (A) Overexpression of miR-382 stimulates doxorubicin- or MTX-induced U2OS cell death, whereas inhibition of miR-382 protected U2OS cells from doxorubicin- or MTX-induced death. U2OS cells were transfected with indicated nucleotides. After 24 hrs of transfection, 4 × 103 cells/well were seeded in 96-well cell culture plates. The next day, cells were incubated with or without the indicated concentration of doxorubicin or MTX for 48 hrs and subsequently subjected to an MTT assay. **(B)** Overexpression of miR-382 increased doxorubicin- or MTX-induced apoptosis in MG63 cells. In contrast, inhibition of miR-382 inhibited doxorubicin- or MTX-induced apoptosis in MG63 cells. MG63 cells were transfected with the indicated nucleotides. After 24 hrs of transfection, cells were seeded into 6-well cell culture plates. The next day, cells were treated with DMSO or doxorubicin/or MTX for 48 hrs, followed by a flow cytometric assay. **(C)** Overexpression of miR-382 increased doxorubicin- or MTX-induced caspase 3 activity, whereas inhibition of miR-382 suppressed doxorubicin- or MTX-induced caspase activity in MNNG/HOS cells. MNNG/HOS cells were transfected with the indicated nucleotides. After 24 hrs of transfection, cells were seeded into 6-well cell culture plates. The next day, cells were treated with DMSO or doxorubicin/or MTX for 48 hrs, and subsequently subjected to caspase 3 activity assay. All data are presented as the mean ± SD from three independent experiments. Ctrl.Oligo, control oligonucleotides; ASO miR-382, miR-382 antisense nucleotides.

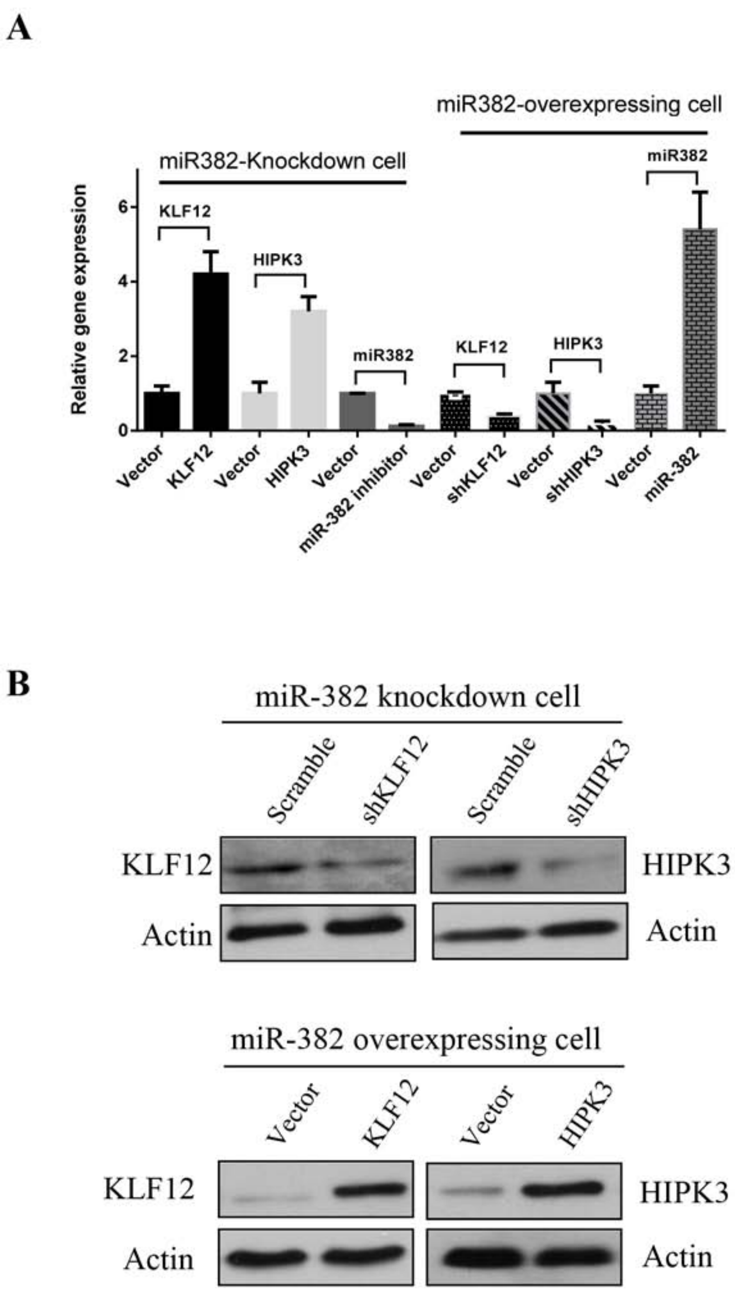

Supplementary Figure S3. Expression of KLF12 and HIPK3. The indicated plasmids were transfected into miR-382-overexpression or -knockdown cell lines and their corresponding mRNA (A) and protein **(B)** expression levels were measured. The data are presented as the mean ± SD from three independent experiments.

Supplementary Table 1. Patient characteristics

	miR-382 Low		miR-382 High		*p*
	**No.**	**%**	**No.**	**%**	
Total patients	51		64		
Gender					0.22
Male	29	56.9	29	45.3	
Female	22	43.1	35	54.7	
Age					0.68
Median	15.9		16.3		
Range	3–40		4–45		
Anatomical site					0.81
Femure	25	49.0	34	53.1	
Tibia	11	21.6	17	26.6	
Humerus	10	19.6	9	14.1	
Pelvis	2	3.9	1	1.6	
Other	3	5.9	3	4.7	
Histologic subtype					0.65
Osteoblastic	29	56.9	41	64.1	
Chondroblastic	6	11.8	9	14.1	
Fibroblastic	11	21.6	7	10.9	
Telangiectatic	3	5.9	4	6.3	
Other	2	3.9	3	4.7	
Histologic grade					0.47
III	7	13.7	12	18.8	
IV	44	86.3	52	81.3	
Ennecking grade					0.89
2a	6	11.8	7	10.9	
2b	45	88.2	57	89.1	

Supplementary Table 2. Univariate and multivariable analyses of factors predictive of poor overall survival in osteosarcoma patients

Variable	Univariate		Multivariable	
	HR (95% CI)	*p* value	HR (95% CI)	*p* value
Gender	0.84(0.46-1.53)	0.56	0.76 (0.41-1.42)	0.39
Age	1.02(0.98-1.05)	0.42	1.03 (0.98-1.07)	0.26
Anatomical site	----	0.87	----	0.94
Histologic subtype	----	0.52	----	0.67
Histologic grade	1.86 (0.73-4.72)	0.20	1.90 (0.69-5.18)	0.21
Ennecking grade	0.63 (0.28-1.43)	0.27	0.71 (0.26-1.96)	0.51
miR-382 level	0.37 (0.15-0.96)	0.04	0.42 (0.16-1.09)	0.07
Chemoresponse	2.43 (1.33-4.44)	0.004	2.18 (1.13-4.19)	0.02
